# Prevalent findings on low-dose CT scan lung cancer screening: a French prospective pilot study

**DOI:** 10.1093/eurpub/ckae183

**Published:** 2024-11-20

**Authors:** Philippe A Grenier, Maxence Arutkin, Anne Laure Brun, Anne-Cécile Métivier, Edouard Sage, Franck Haziza, Félix Ackermann, François Mellot, Alexandre Vallée

**Affiliations:** Department of Clinical Research and Innovation, Foch Hospital, Suresnes, France; Department of Epidemiology and Public Health, Foch Hospital, Suresnes, France; Department of Radiology, Foch Hospital, Suresnes, France; Department of Pneumology, Foch Hospital, Suresnes, France; Department of Thoracic Surgery, Foch Hospital, Suresnes, France; Department of Cardiology, Foch Hospital, Suresnes, France; Department of Internal Medicine, Foch Hospital, Suresnes, France; Department of Radiology, Foch Hospital, Suresnes, France; Department of Epidemiology and Public Health, Foch Hospital, Suresnes, France

## Abstract

Despite significant therapeutic advances, lung cancer remains the biggest killer among cancers. In France, there is no national screening program against lung cancer. Thus, in this perspective, the Foch Hospital decided to implement a pilot and clinical low-dose CT screening program to evaluate the efficiency of such screening. The purpose of this study was to describe the prevalent findings of this low-dose CT screening program. Participants were recruited in the screening program through general practitioners (GPs), pharmacists, and specialists from June 2023 to June 2024. The inclusion criteria included male or female participants aged 50 to 80 years, current smokers or former smokers who had quit less than 15 years prior, with a smoking history of over 20 pack-years. Chest CT scans were conducted at Foch Hospital using a low-dose CT protocol based on volume mode with a multi-slice scanner (≥60 slices) without contrast injection. In total, 477 participants were recruited in the CT scan screening, 235 (49%) were males with a median age of 60 years [56–67] and 35 smoke pack-years [29–44] and 242 females (51%) with a median age of 60 years [55–60] and 30 smoke pack-years [25–40]. Eight participants showed positive nodules on CT scan, as a 1.7% rate. 66.7% of diagnosed cancers were in early stages (0-I). It is feasible to implement structured lung cancer screening using low-dose CT in a real-world setting among the general population. This approach successfully identifies most early-stage cancers that could be treated curatively.

## Introduction 

Despite significant therapeutic advances made over the past 10 years, lung cancer remains the biggest killer among cancers (33 000 deaths in 2018). The lung is an organ without sensory innervation where cancer develops silently. When symptoms appear, it is generally too late to propose curative treatment. Contrary to widespread belief, the growth of lung cancer is not explosive. The average duration of the pre-clinical phase (i.e. the period between stage IA, where the cancer becomes visible on a scan, and stage IIIA, considered the last operable stage) is estimated to be between 3 and 4 years for non-small cell carcinomas, which corresponds to a window of opportunity for detecting cancer at an asymptomatic stage [1].

There is therefore a rationale for lung cancer screening, but its effectiveness still needs to be demonstrated. This has now been achieved, notably through two large-scale randomized prospective studies—National Lung Screening Trial (NLST) and Nelson—which showed a reduction in specific mortality of 20% and 26%, respectively [2, 3], with a benefit likely even more pronounced in women. It can therefore be considered that the scientific effectiveness of screening has been demonstrated, and this constitutes a crucial step [1]. This was recently confirmed by Henscke *et al.* [4]. Among 89 404 participants in the annual low-dose CT scan screening of the international ELCAP (Early Lung Cancer Action Program) group, 1257 (1.4%) had primary lung cancer [4]. After 20 years of follow-up, cancer cure could be confirmed in 81% of cases. This figure compares to the 16% cure rate when cancer diagnosis is made outside a screening program [1].

Numerous low-dose CT scan screening programs are already implemented in the USA. They follow the new United States Preventive Services Task Force (USPSTF) recommendations, which recommend screening for smokers aged 50–80 years with a cumulative smoking history of at least 20 pack-years [5]. Given the success of the pilot study “UK Lung Cancer Screening Trial,” a national program has just been established in Great Britain [6]. In Europe, organized screening programs or pilot studies are already operational in several European countries [7, 8]. In France, a group of French experts, pulmonologists, and radiologists recently proposed a comprehensive update on low-dose CT scan lung cancer screening [8]. These experts resolutely favor individual screening as already practiced, but especially push for large-scale experiments in pilot territories to demonstrate the feasibility of mass screening on a national scale. There is indeed urgency, as it is estimated that organized screening in France could prevent between 2200 and 7400 deaths per year [9].

In France, there is no national screening program against lung cancer. Finally, last year, under the impetus of Emmanuel Macron, the President of the French Republic, the Haute Autorité de Santé (French National Authority for Health) entrusted the mission to INCa (National Institute for Cancer) to implement a national lung cancer screening program by low-dose CT scan for at-risk individuals. INCa has decided to plan a national pilot study while not prohibiting any initiative that would contribute to enriching the discussion.

Thus, in this perspective, the Foch Hospital, Suresnes, France, decided to implement a pilot and clinical low-dose CT screening program to evaluate the efficiency of such screening. The purpose of this study was to describe the prevalent findings of this low-dose CT screening program.

## Methods

**Figure 1.**
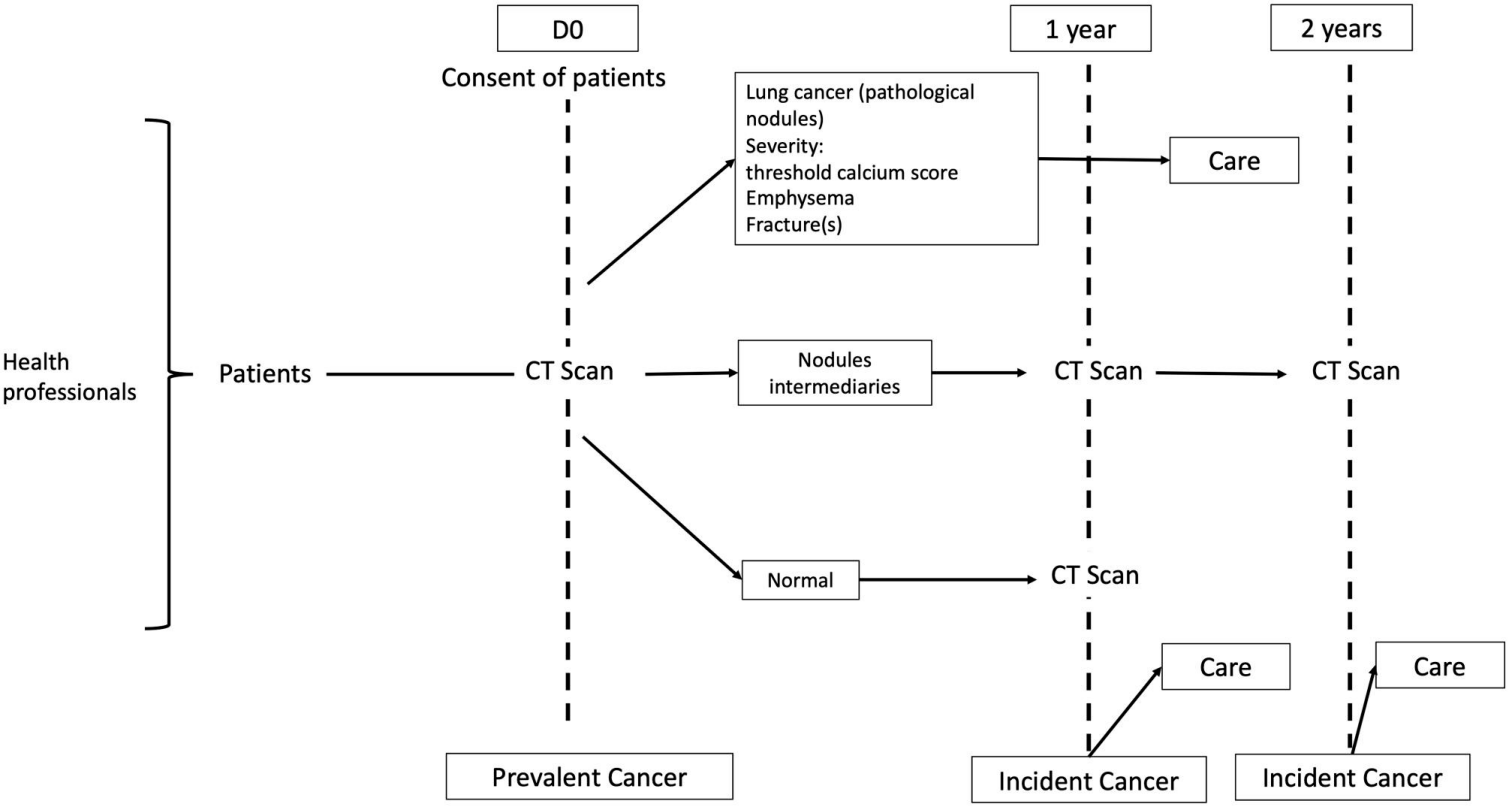
Description of the low-dose CT screening program.

Participants were recruited in the screening program through general practitioners (GPs), and specialists from June 2023 to June 2024 (Fig. 1). The inclusion criteria included male or female participants aged 50–80 years, current smokers or former smokers who had quit less than 15 years prior, with a smoking history of over 20 pack-years. Participants needed to be symptom-free, informed about various smoking cessation aids, and willing to accept the screening methods after being clearly informed about the potential results and consequences (non-invasive procedures: chest CT at 3 months, positron emission tomography [PET]; invasive procedures: CT-guided biopsy, bronchial fibroscopy, exploratory surgery, etc.).

Exclusion criteria included a history of cancer within the last 5 years, significant health status deterioration.

Health professionals in the Hauts de Seine administrative area in France were invited to participate via letter. Doctors were offered training and awareness sessions and were engaged through medical educational meetings and post-doctorate lectures. Public information campaigns included press articles, radio and TV broadcasts, posters, and leaflets.

Physicians and radiologists participating verified the inclusion and exclusion criteria. They explained the screening protocol and informed each patient about the possibility of detecting abnormalities with CT and the potential need for additional procedures. Each consultation also provided an opportunity to offer smoking cessation advice or referrals to anti-smoking programs.

Patients received written information about the screening methods, expected benefits, and potential drawbacks.

Chest CT scans were conducted at Foch Hospital using a low-dose CT protocol based on volume mode with a multi-slice scanner (≥60 slices) without contrast injection. The nominal slice thickness was <1.5 mm, with iterative reconstructions. Subjects were placed in a supine position with their arms above their head, and a spiral acquisition of the entire chest was performed from the apexes to the inferior pleural recesses during a deep breath hold. The low-dose radiation technique conformed to international recommendations, with dose-length product (DLP) parameters adapted to weight (150–180 mGy/cm), a maximum DLP for a standard subject of 150 mGy/cm, and a target value of 100 mGy/cm. Acquisition parameters ranged from 100 to 140 kV and from 40 to 50 mAs, depending on the subject’s weight.

A double reading interpretation of low-dose CT scans by radiologists was organized using a standardized report. The final report was sent to the coordinating center and the appropriate referent physician (GP or pulmonologist) who then managed treatment based on predefined algorithms for solid, subsolid, or ground glass nodules. If no suspicious nodules were detected, the screening was considered negative, and the subject was given an appointment for the next annual low-dose CT screening. For nodules >10 mm, the screening was positive, and the patient’s records were reviewed at a multidisciplinary team meeting (MDTM). For nodules between 5 and 10 mm, the screening was undetermined, and an intermediary low-dose CT was performed 3 months later. Lung cancer diagnosis was established based on anatomopathological analysis of surgical samples or biopsies. The WHO lung cancer classification was applied [10]. The stage was defined using the International Association for the Study of Lung Cancer Staging Guidelines (8th ed.) [1].

The severity of emphysema was judged visually according to the Fleischner Society classification [11], and the calcium score was established on the four coronary arteries (common trunk, anterior interventricular, right coronary, and circumflex) [12]. The measurement of bone density at the center of the vertebrae on a chest CT is correlated with that obtained by DXA (dual-photon absorptiometry) [13]. A density measurement value of less than 100 HU on a low-dose CT scan is a reliable criterion for the diagnosis of osteoporosis [14]. Care pathways were predefined for subjects with severe emphysema, high coronary calcium score, and osteoporosis.

Finally, incidental findings, such as interstitial lung disease, significant airway disease, mediastinal abnormalities, were noted, and the patients were referred to the pulmonologist.

The study was approved by the Foch IRB: IRB00012437 (approval number: 24-04-01) on 10 April 2024. Consent was obtained for all participants.

### Statistical analysis

Continuous variables were presented with median (25th–75th percentile) and with number and percentage for categorical variables. Comparison between gender groups was performed with Mann–Whitney *U* test for continuous variables and with Fisher’s test or chi-square test for categorical variables. Analyses were performed with SAS software (version 9.4; SAS Institute, Carry, NC, USA).

## Results

**Figure 2.**
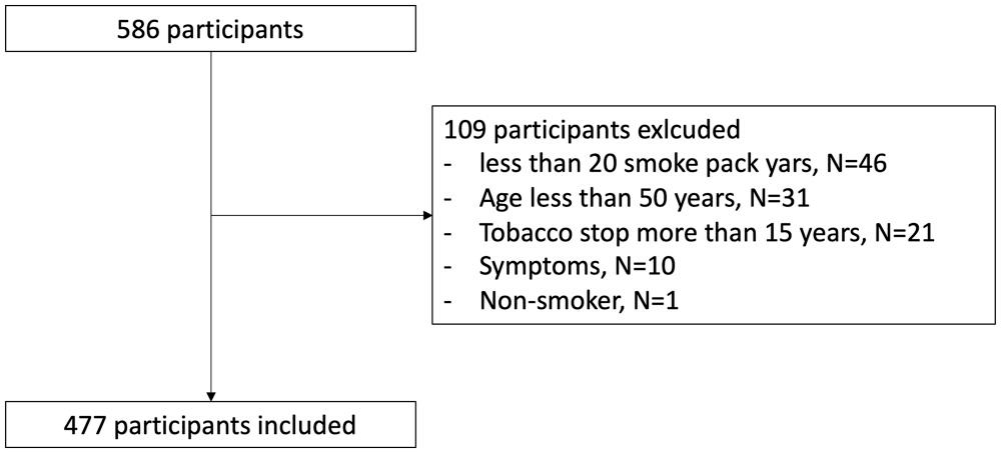
Flowchart.

In total, 586 participants were recruited in the Hospital, but only 477 were included in the CT screening program, 109 participants were excluded due to exclusion criteria (under 20 smoke pack-years especially; Fig. 2). One participant among the excluded participants showed a positive nodule.

About 235 (49%) were males with a median age of 60 years [56–67] and 35 smoke pack-years [29–44] and 242 females (51%) with a median age of 60 years [55–60] and 30 smoke pack-years [25–40]. Eight participants showed positive nodules on CT scan, as a 1.7% rate. Four males (1.7%) presented a positive result of nodule on CT scan, and four females (1.7%) were positive. Six (2.6%) males and 17 (7.0%) females were intermediates. About 175 males (75.8%) and 213 females (88.0%) presented no emphysema, 203 males (86.8%) and 191 (78.9%) females presented no osteoporosis, while only 70 (30.7%) males and 101 (41.9%) of females presented no coronary damages (Table 1). 66.7% of diagnosed cancers were in early stages (0-I; Supplementary Table S1). One nodule was related to a renal cell carcinoma metastasis, the renal lesion was diagnosed simultaneously. We observed no difference between males and females for the prevalence of positive nodules (*P* = 0.966). The screening adherence 12 months after baseline scan was 45% (49 patients of the 108 eligibles at one year).

**Table 1.** Descriptive table of the study population

	Females		Males		*P*-value
Age	60	[55–60]	60	[56–67]	0.646
Smoke pack-years	30	[25–40]	35	[29–44]	0.041
Nodule					0.074
Intermediate	17	7.0%	6	2.6%	
Negative	221	91.3%	225	95.7%	
Positive	4	1.7%	4	1.7%	
Emphysema					<0.001
Confluent CLE	8	3.3%	7	3.0%	
Advanced destructive E	1	0.4%	4	1.7%	
Moderate CLE	20	8.3%	46	19.7%	
Mild CLE	0	0.0%	2	0.9%	
Trace or negative	213	88.0%	175	74.8%	
Coronary calcium score					<0.001
Severe (6 or more)	11	4.6%	24	10.5%	
Moderate (4 or 5)	33	13.7%	57	25.0%	
Low (1–3)	96	39.8%	77	33.8%	
Absence	101	41.9%	70	30.7%	
Osteoporosis					0.071
Negative	191	78.9%	203	86.8%	
Osteoporosis	38	15.7%	22	9.4%	
Osteoporosis and compaction fracture	13	5.4%	9	3.8%	

Characteristics of participants showing positive nodules were presented in Supplementary Table S1.

## Discussion

Lung cancer is most frequently diagnosed in people over the age of 65, with the median age at diagnosis being 70. Despite this, lung cancer can develop at any age, and there has been a recent rise in cases among younger individuals who have never smoked. Lung cancer screening involves using imaging tests to detect the disease before symptoms arise. The primary aim is to identify lung cancer at an early stage, making it easier to treat and potentially cure. The most widely used screening method is low-dose CT scan. This imaging technique uses minimal radiation to capture detailed images of the lungs and has proven effective in spotting lung cancer when it is still localized and has not metastasized [15].

One of the key benefits of this screening test is its ability to detect lung cancer at an early stage. Early detection allows for more effective treatment and significantly improves survival rates. Additionally, low-dose CT scan lung cancer screening is non-invasive, reducing the number of needed biopsies or other invasive diagnostic procedures, thus making the process more comfortable for patients. The screening is also quick and painless, typically taking less than 10 min to complete. Furthermore, lung cancer screening scans can identify individuals with evidence of previously undiagnosed cardiovascular disease, emphysema, or osteoporosis, and provide an opportunity for treatment or prevention [16].

In North America, both the US Preventive Services Task Force and the Canadian Task Force on Preventive Health Care recommend this technique for individuals aged 55 and older who are smokers or former smokers who quit within the last 15 years, with a smoking history of over 30 pack-years [17]. In Europe, the European Respiratory Society recommended in 2015 that this screening method be adopted either as part of a research program or in certified centers as standard clinical practice. These centers should include radiologists, pulmonologists, oncologists, pathologists, thoracic surgeons, and tobaccologists [18]. Thus, as recently as May 2016, the French National Authority for Health (HAS) stated that the conditions required to implement this screening approach in France had not yet been met. This is why we decided to implement the number of smoke pack-years to 20 in our study, as observed in the study ELCAP [4].

Our study demonstrates the success of such implementation of CT screening program with 1.7% positive nodules diagnosed. The prevalence of lung cancer in our study was 1.7%, lower to the 2.7% of the previous French study [8], the 2.6% rate in NELSON [16], 2.4% by NLST [5], 2.4% in DEPISCAN [17], and 2.2% by DANTE [18], but superior to the 1.5% reported by ITALUNG [19] and to the 1.4% reported by the ELCAP group [4]. In our study, 66.7% of diagnosed cancers were in early stages (0-I), in line with other studies: 67.7% in the previous French study [8], 63% in Stage I in NLST [5], 66% in NELSON [19], 65.7% in ITALUNG [20], 63.6% in DANTE [21], and over 37.5% in Stage I in DEPISCAN [22]. Oncological surgery is still the fundamental curative treatment for localized stages and our lung cancers detected were treated by surgical lung resection, the majority involving limited resections. One wedge resection, one segmentectomy, one sub-lobar pluri-segmentectomy, one sub-lobar atypical resection, one lobectomy, and 0 pneumonectomy were performed.

The surgical resection rate is inferior to the 80% rate reported for ITALUNG [20] and 75% for the PanCAn study [23] while being comparable to the 61% for NLST [5] and 65% for the Lung Health Check study [24]. This is one of the efficacy markers for screening method, as surgery actually results in over 80% 5-year survival rates in localized stage cancer [25].

To ensure equitable access to lung cancer screening, it is vital to identify and address existing disparities in healthcare availability. Efforts should focus on reaching high-risk populations, particularly those from socioeconomically disadvantaged and marginalized communities. Implementing strategies such as community outreach, mobile screening units, and targeted awareness campaigns can significantly improve access to screening for these underserved groups [26].

In our study, the screening adherence 12 months after baseline scan was 45% (49 patients on the 108 eligibles at one year). This score is superior to the observed adherence rate on previous studies, around 30% (95% CI: 18%–44%) [24]. In fact, a coordinator is specifically dedicated to our screening program to send reminder emails for this second scan and, if necessary, or to call patients directly to schedule an appointment.

Comprehensive patient education is crucial for promoting shared decision-making in lung cancer screening. Patients need clear and understandable information about the benefits and risks of screening, including the potential for false positives and overdiagnosis. Education should also highlight the importance of smoking cessation and incorporate cessation programs alongside screening initiatives. By engaging patients in shared decision-making, they are empowered to make informed choices that reflect their preferences and values [27].

The economic and political implications of a national lung cancer screening program in France are profound, considering both the associated costs and the potential for significant mortality reduction [6]. Drawing from the Foch Hospital’s pilot findings and outcomes in countries with established screening programs, such as the USA, the UK, and various European nations, a national program could potentially decrease lung cancer mortality by 20%–26% by identifying early-stage cancers [3, 26]. Economically, implementing such a program entails substantial initial costs, particularly in setting up screening infrastructure and maintaining yearly low-dose CT scans for at-risk populations. In France, where annual lung cancer deaths number around 33 000 [27], the screening program could save between 2200 and 7400 lives per year, which in the long term may reduce treatment costs associated with advanced lung cancer. The experiences of other countries underline these potential benefits among high-risk populations (cost-effectiveness was optimal in those aged 55–75 years and smoking history of at least 20 pack-years) [28]. For instance, the USA has found that systematic screening reduces not only lung cancer mortality but also long-term treatment costs by shifting from high-cost late-stage treatments to early-stage, potentially curative interventions [29, 30]. Similarly, the European recent national screening initiatives suggested a high acceptance rate among at-risk populations and have projected positive economic returns from early detection and treatment [31–34]. Implementing a comparable national program in France could yield similar benefits, but policymakers would need to address logistical issues such as equitable access, program awareness, and sustainable funding.

### Limitations

The main limitation of this monocentric pilot study is the relatively low number of subjects recruited during the first year of screening. Extensive efforts remain to be made to enlarge the program deployment area. The recruitment was mainly done through the GPs working in the surroundings of the Hospital. The recruitment could be increased by sharing the same program with other hospitals that would agree to make information campaign for GPs in their area. A selection bias is unavoidable. The participants are those mostly motivated and often those with easier access to the health care system. In case of larger deployment, a certain amount of equity is recommended by increasing dedicated resources in the economically and socially disadvantaged areas. Smoking history was assessed firstly by the GPs in charge to recruit the participants. Then, smoking history was re-assessed by the Department of Radiology at the time of inclusion. This double-check limits the assessment errors but does not avoid the risk of self-overestimation by a participant willing not being excluded from inclusion. Smoking history was assessed firstly by the GPs in charge to recruit the participants. Then, smoking history was re-assessed by the Department of Radiology at the time of inclusion. This double-check limits the assessment errors but does not avoid the risk of self-overestimation by a participant willing not being excluded from inclusion.

The lack of artificial intelligence (AI) support to improve the radiologists’ performances was the second limitation of our study. AI demonstrates significant potential in improving nodule detection sensitivity, reducing false-positive rates, and classifying nodules, while also showing value in predicting nodule growth and pathological/genetic typing. can potentially increase the efficiency of lung cancer screening (LCS) [35]. Some other AI algorithms can segment automatically the lungs and lobes on CT images, quantify emphysema by densitometry, calculate the volume of calcifications in the coronary arteries, and measure the density and height of thoracic vertebra to detect osteoporosis and compression fractures. However, the radiologists’ performances in detecting pulmonary nodules with and without AI solutions must be assessed and concordances between radiologists reading screening CT scans with and without AI support have to be compared. Such evaluations will be necessary to validate the potential replacement of double readings by a single reading associated with an AI solution.

## Conclusion

Our research shows that it is feasible to implement structured lung cancer screening using low-dose CT in a real-world setting among the general population. This approach successfully identifies most early-stage cancers that could be treated curatively.

## Supplementary Material

ckae183_Supplementary_Data

## Data Availability

Data cannot be shared publicly because of they are the property of Foch Hospital. Data are available from the Clinical Research Center of the Foch Hospital for researchers who meet the criteria for access to confidential data. Key points In France, there is no national screening program against lung cancer. In our study, eight participants showed positive nodules on CT scan, as a 1.7% rate. 66.7% of diagnosed cancers were in early stages (0-I). It is feasible to implement structured lung cancer screening using low-dose CT in a real-world setting among the general population. This approach successfully identifies most early-stage cancers that could be treated curatively. Screening adherence 12 months after baseline scan was 45%.
