# Correction to: Prevalent findings on low-dose CT scan lung cancer screening: a French prospective pilot study

**DOI:** 10.1093/eurpub/ckaf050

**Published:** 2025-06-02

This is a correction to: Philippe A Grenier, Maxence Arutkin, Anne Laure Brun, Anne-Cécile Métivier, Edouard Sage, Franck Haziza, Félix Ackermann, François Mellot, Alexandre Vallée, Prevalent findings on low-dose CT scan lung cancer screening: a French prospective pilot study, *European Journal of Public Health*, 2024; ckae183, https://doi.org/10.1093/eurpub/ckae183

In the originally published version of this manuscript, two references were erroneously omitted and a number of references were wrongly positioned. These errors have been corrected.

