# Supplementary material for: Prevalent findings on low-dose CT scan lung cancer screening: a French prospective pilot study
Source: Eur J Public Health. 2024 Nov 20;35(2):342–6. doi: 10.1093/eurpub/ckae183 (PMC11967878; doi:10.1093/eurpub/ckae183)
Supplement: ckae183_Supplementary_Data [file ckae183_supplementary_data.docx]

**Table S1: Description of participants with positive nodule**

| **Age** | **Gender** | **Smoke pack years** | **Nodule** | **Emphysema** | **Coronary Calcifications Score** | **Osteoporosis** | **Pathology and Stage of nodule** | **Care / surgery** | **Classification and survival** |
| --- | --- | --- | --- | --- | --- | --- | --- | --- | --- |
| 62 | Female | 40 | Positive | No | Low | Yes | Mini-invasive pulmonary adenocarcinoma (80% lepidic and 20% acinar) 15 mm diameter with 5 mm solid component: T1min N0 M0 (Stage IA) | Upper segment of LLL segmentectomy | TP  alive |
| 67 | Male | 42 | Positive | Moderate | Severe | No | Adenocarcinoma (papillary /acinar) in the LLL: T1b N0 M0 (Stage IA) and Adenocarcinoma (papillary) in the LUL: T1a N0 M0 (Stage IA) | LLB basilar segmentectomies  Nodule wedge resection in the LUL | TP  alive  TP |
| 69 | Male | 53 | Positive | Moderate | Low | No | Small cell lung cancer: T4 N2 M1 (Stage IV) | Chemotherapy + Radiation therapy | TP  alive |
| 75 | Female | 100 | Positive | No | Moderate | Yes | Squamous cell carcinoma: T2a N0 M0 (Stage IB) | Pulmonary fibrosis  No surgical resection so far | TP  alive |
| 63 | Female | 47 | Positive | Confluent | Low | Fracture | Squamous cell carcinoma (9 mm) in the LLL: T1a N0 M0 (Stage IA) | Atypical resection of the left lower lobe | TP  alive |
| 59 | Male | 50 | Positive | Severe | No | No | Squamous cell Carcinoma: T4 N2 M1 (Stage IV) | Chemotherapy and immunotherapy | TP  alive |
| 76 | Female | 20 | Positive | Moderate | Low | Yes | Adenocarcinoma (lepidic)  30 mm part-solid nodule in the RUL  T1c N0 M0 (Stage IA) | Lobectomy of RUL | TP  alive |
| 70 | Male | 50 | Positive | Moderate | Severe | No | Adenocarcinoma (papillary) : T1 N2 M0 (Stage IIIA) | Inoperable patient because of severe coronary disease | TP  alive |
